# Progranulin-Derived Atsttrin Directly Binds to TNFRSF25 (DR3) and Inhibits TNF-Like Ligand 1A (TL1A) Activity

**DOI:** 10.1371/journal.pone.0092743

**Published:** 2014-03-20

**Authors:** Cui Liu, Xing-Xia Li, Wei Gao, Wen Liu, De-Shan Liu

**Affiliations:** 1 Department of Pediatric Surgery, Qilu Hospital of Shandong University, Jinan, China; 2 Department of Nursing, Qilu Hospital of Shandong University, Jinan, China; 3 Department of Biotechnology, Taishan Medical University, Taian, China; 4 Department of Traditional Chinese Medicine, Qilu Hospital of Shandong University, Jinan, China; French National Centre for Scientific Research, France

## Abstract

Atsttrin, a progranulin (PGRN)-derived molecule composed of three TNFR-binding domains of PGRN, binds to TNF receptors (TNFR) and is therapeutic against inflammatory arthritis. Here we screened the associations of Atsttrin and other members in TNFR subfamily, which led to the discovery of TNFRSF25 (DR3) as an additional Atsttrin-interacting member in TNFR family. Similar to TNFR1 and TNFR2, DR3 also directly bound to Atsttrin. The first three cysteine-rich domains (CRD) in the extracellular portion of DR3 were required for this interaction. Atsttrin inhibited the interaction between DR3 and its TNF-Like Ligand 1A (TL1A). In addition, Atsttrin inhibited TL1A-stimulated target gene expressions and neutralized TL1A-enhanced osteoclastogenesis in vitro. Furthermore, Atsttrin ameliorated the pathology in dextran sulfate sodium induced colitis. Taken together, these findings not only provide the new insights into Atsttrin's therapeutic action in inflammatory arthritis, but may also present Atsttrin as a novel biological agent for treating various types of diseases associated with TL1A/DR3 pathway.

## Introduction

Progranulin (PGRN) is a growth factor with multiple biological functions including anti-inflammation and immune regulations [1]. PGRN contains seven-and-a-half repeats of a cysteine-rich motif (CX_5–6_CX_5_CCX_8_CCX_6_CCXDX_2_HCCPX_4_CX_5–6_C) in the order P-G-F-B-A-C-D-E, where A-G are full repeats and P is the half-motif [2]. PGRN was reported to bind to TNF receptors (TNFR) through three individual and separate binding domains involving granulin A, C and F plus adjacent linkers [3]. Atsttrin (Antagonist of TNF/TNFR Signaling via Targeting to TNF Receptors) is an engineered molecule composed of half units of granulins A, C and F plus linkers P3, P4 and P5 that appears to be the “minimal” engineered molecule retaining affinity to TNFR [3]–[5]. Atsttrin was reported to selectively bind to TNFR and inhibited the binding of TNFα to TNFR in vitro. In addition, recombinant Atsttrin protein effectively attenuated inflammation in several animal models, including collagen antibody- and collagen-induced arthritis models, TNF transgenic mice and dermatitis model [3], [6], indicating that Atsttrin may represent a novel biologics for treating various kinds of TNF/TNFR associated inflammatory diseases and conditions [3]–[5], [7].

TNF and TNFR superfamilies (TNFSF and TNFRSF) consist of approximately 50 membrane and soluble proteins that can modulate cellular function [8]. Receptors are usually type I and sometimes type III membrane proteins, and characterized by the presence of one to four cysteine-rich domains (CRD) in their extracellular portion. Most of these molecules have a wide range of actions including promoting cellular differentiation, survival, and production of inflammatory cytokines and chemokines. Experimental and genetic evidences have demonstrated that TNFSF ligand–receptor signaling pathways are active in inflammatory and autoimmune diseases. Targeting these pathways has been proven to be highly successful for treatment of several autoimmune diseases including rheumatoid arthritis and Crohn's disease [9], [10].

Death Receptor 3 (DR3), also known as TNFRSF25, TRAMP, LARD, or WSL-1, is a death-domain-containing TNF family receptor, shows the highest homology to TNFR1 [11]–[13]. However, unlike TNFR1, which is ubiquitously expressed, DR3 has been reported to be expressed primarily by T lymphocytes [14]–[16]. TL1A was identified as the only known and confirmed ligand for DR3 [17]. Like other TNF members, TL1A contains a predicted transmembrane domain and a bioactive, proteolytically cleaved truncated form that can be released as a soluble factor [18]–[20]. TL1A expression is highly induced by inflammatory stimuli, such as lipopolysaccharide and Fc-receptor crosslinking in macrophages and dendritic cells, as well as other inflammatory cytokines such as IL-1 and TNF in endothelial cells [14], [18], [21], [22]. TL1A/DR3 interactions are involved in the development of diverse autoimmune diseases, as demonstrated in inflammatory bowel disease and in experimental models such as chronic murine ileitis and experimental autoimmune encephalomyelitis [23]. Blockade of TL1A/DR3 interactions strikingly reduces pathology in a number of animal models [14].

Although it is known that some TNFL/TNFR interactions are mutually exclusive, cross-interactions have been reported in a majority of cases [24], [25]. For example, LTα mediates a variety of inflammatory, immunostimulatory, and antiviral responses through binding to several members in TNFR family, including TNFR1, TNFR2 and HVEM [25]. Here we report that in addition to TNFR1 and TNFR2, Atsttrin also directly binds to DR3 and inhibits TL1A binding and activity.

## Results

### Atsttrin selectively binds to TNFR and DR3 among TNFR super family

**Figure 1:**
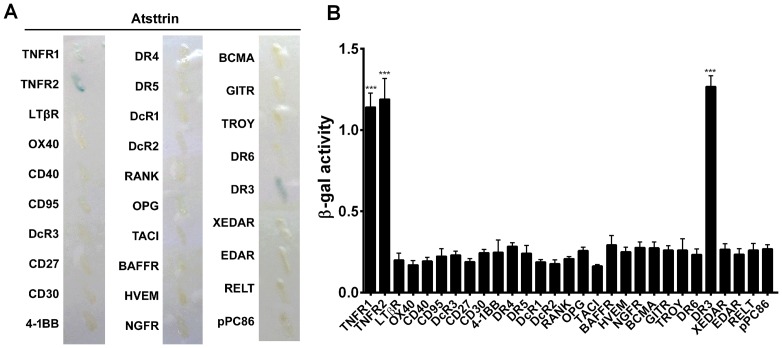
Atsttrin specifically binds to TNFR and DR3 in TNFR super family. A. cDNA encoding Atsttrin was fused to the Gal4 DNA binding domain in the pDBLeu vector and cDNAs encoding the extracellular portions of 28 TNFRSF members were fused to the Gal4 activation domain in the pPC86 vector. Selected plasmids were co-transformed into yeast strain MAV203; X-Gal assay was performed to determine β-galactosidase phenotype. B. Quantitative assay for β-galactosidase (β-gal) activity in liquid cultures of the interactions between Atsttrin and TNFR superfamily members were determined using o-nitropenyl-β-D-galactopyranoside (ONPG) as a substrate. For each strain, three independent colonies were analyzed and triplicate samples for each colony. pPC86 empty vector was used as a negative control. ***p<0.001 versus negative control.

The previous finding that Atsttrin bound to TNFR1 and TNFR2 [3], and the recent report that PGRN bound to the 2^nd^ and 3^rd^ cysteine rich domain (CRD) of the extracellular portion of TNFR [26], promoted us to determine whether Atsttrin also associated with other members in TNFR superfamily. For this purpose, we cloned the extracellular portions of all 28 TNFR super family members which have CRD in their extracellular potion, with the exception of Fn14, and tested their interaction with Atsttrin which was cloned into another yeast expression plasmid, using yeast two-hybrid system. We excluded Fn 14 because its extracellular portion does not have CRD [27]. This screen led to the isolation of DR3, a death-domain-containing TNF family receptor, also known as TNFRSF25, TRAMP, LARD, or WSL-1, which shows the highest homology to TNFR1 [11]–[13], as a novel member in TNFR family that also interacted with Atsttrin (Fig. 1A). In addition, the interaction between Atsttrin and DR3 was also measured and compared using quantitative assay for β-gal activity in liquid culture using ONPG as substrate (Fig. 1B).

### Atsttrin directly binds to DR3

**Figure 2:**
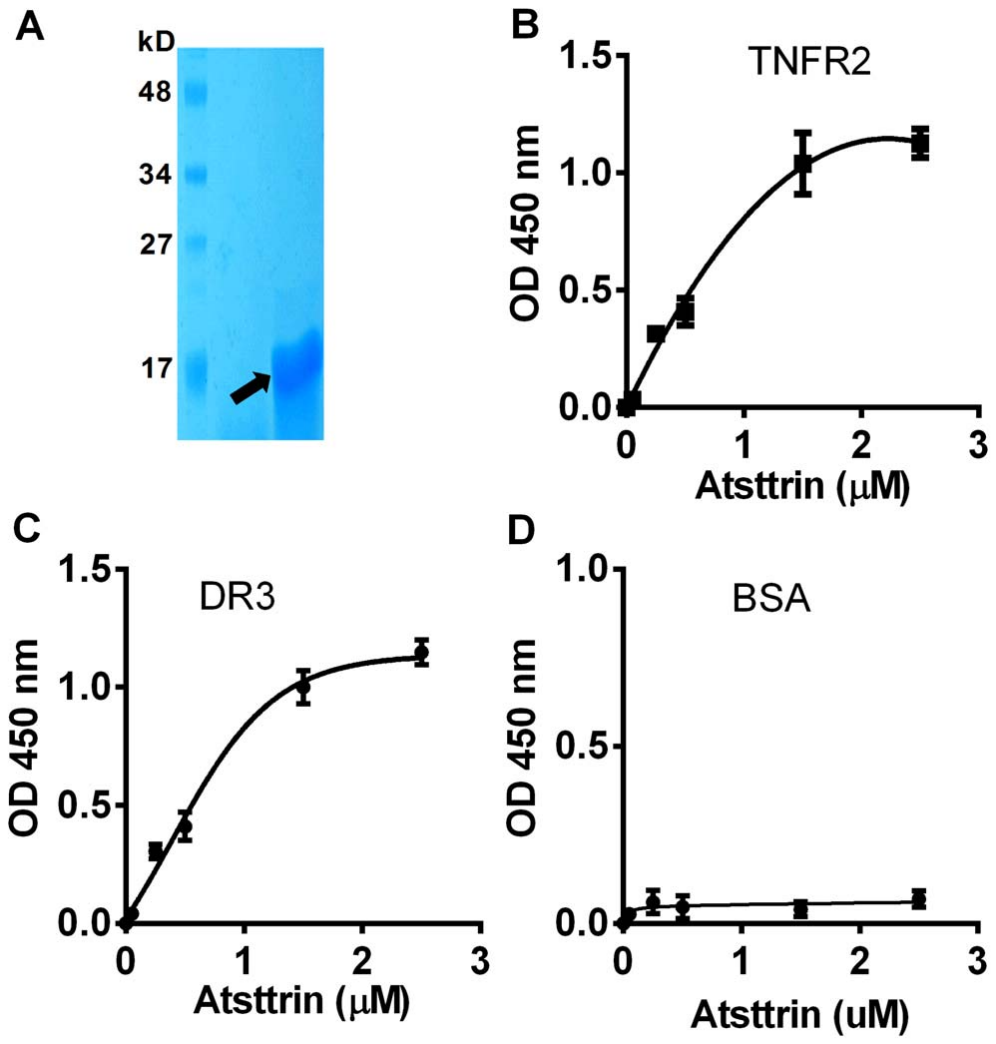
Atsttrin directly binds to TNFR2 and DR3, examined by solid phase binding assay. A. SDS-PAGE analysis of purified Atsttrin. Atsttrin was indicated by an arrow. B and C. Solid phase binding assay. Various dose of Atsttrin was coated to ELISA plate, biotinylated TNFR2 (B), DR3 (C) or BSA (D) was then added to each well, bound protein was detected by adding avidin-HRP to each well and the absorbance was measured at OD 450 nm.

To confirm the binding of Atsttrin to DR3 identified by yeast two-hybrid assay, we next performed solid phase binding assay using recombinant proteins. First we followed published protocol [3] to express and purify the recombinant Atsttrin, and the quality of produced Atsttrin was examined using SDS-PAGE (Fig. 2A). Various dosages of Atsttrin were then coated on a plate and the binding of TNFR2, known to bind to Atsttrin and used as a positive control [3], BSA serving as a negative control, and DR3 were determined. As shown in Fig. 2C, Atsttrin showed dose-dependent binding with DR3, similar to the interaction of Atsttrin with TNFR2 (Fig. 2B). In addition, this assay with only consisted of purified proteins, i.e. purified Atsttrin and DR3 extracellular portion, clearly demonstrated that the interaction between Atsttrin and DR3 is direct. Noted that no interaction between BSA and Atsttrin was detected (Fig. 2D).

### The first three cysteine rich domains of the extracellular potion of DR3 are required for interacting with Atsttrin

**Figure 3:**
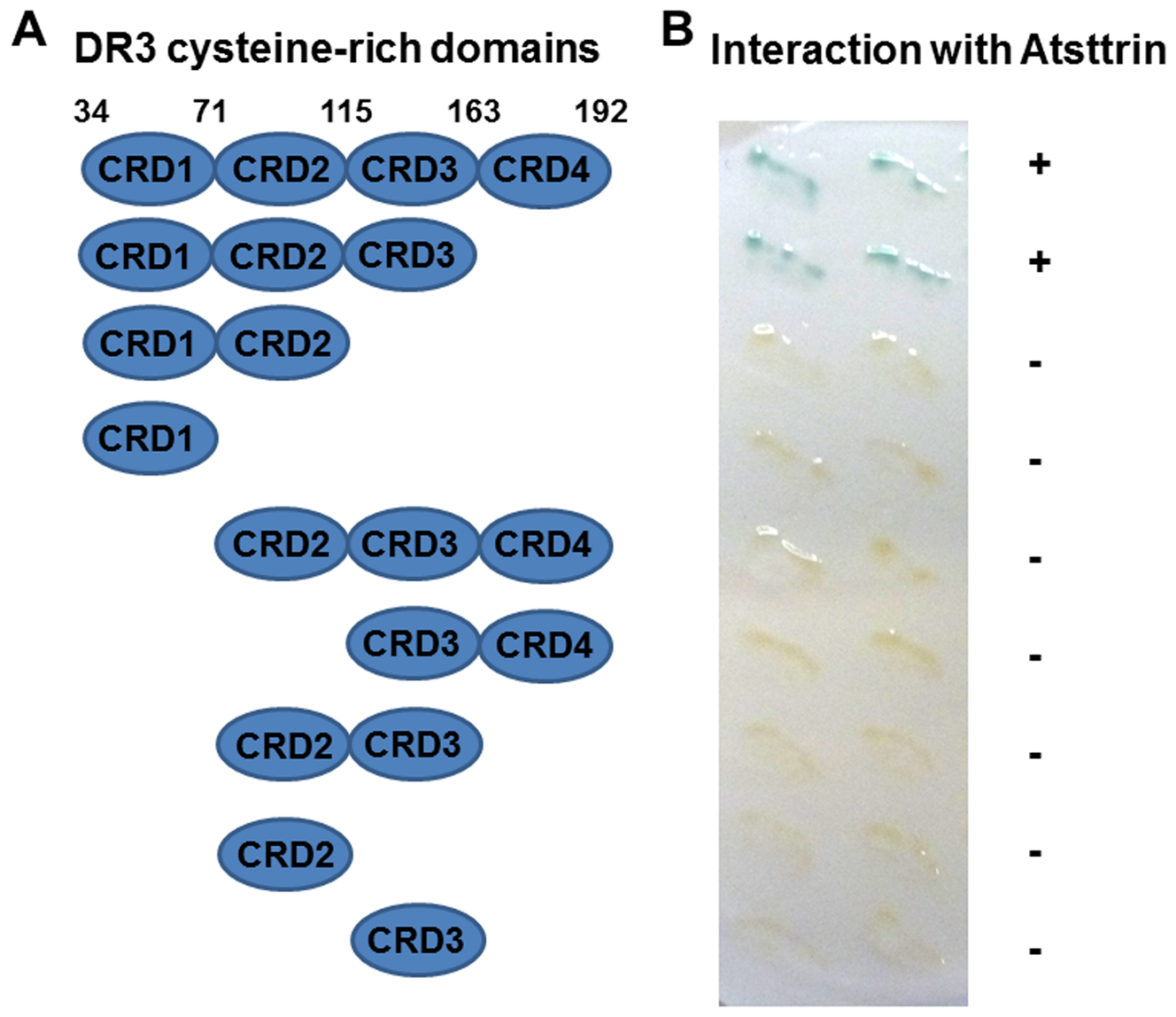
The first three CRDs of DR3 extracellular portion are required for binding to Atsttrin. **A**. Schematic diagram of deletion constructs of DR3 extracellular portion used to map those fragments that bind to Atsttrin. Numbers refer to amino acid residues in the DR3. Interactions between Atsttrin and DR3 derivatives are summarized and indicated by “+” or “−”. **B**. β-Galactosidase activity was used to test interaction between various compositions of extracellular domains of DR3, as indicated, and Atsttrin. Two independent yeast transformants for each pair of plasmids were transferred onto a nitrocellulose membrane and the β-galactosidase activity was determined.

Similar to TNFR, the extracellular potion of DR3 also contains four cysteine rich domains (CRD), CRD1, CRD2, CRD3 and CRD4. To identify which domain(s) are responsible for the interaction between Atsttrin and DR3, we generated various mutants composed of different CRD(s) of DR3, and examined their interaction with Atsttrin. As shown in Fig. 3, removal of CRD4 from C-terminus of the extracellular portion did not affect the interaction with Atsttrin, indicating that CRD4 was not involved in the association with Atsttrin; however, deletion of CRD3 completely abolished the binding, demonstrating that this domain was essential for interacting with Atsttrin. Deletion of CRD1 from N-terminus of the extracellular portion also abolished the interaction. In addition, CRD2 plus CRD3 of TNFR were known to be able to bind PGRN [26], but CRD2 plus CRD3 of DR3 were unable to interact with Atsttrin. Taken together, this set of experiments demonstrated that the first three CRDs, i.e. CRD1, CRD2 and CRD3, of DR3 extracellular portion were all required for its binding to Atsttrin.

### Atsttrin inhibits the interaction between TL1A and DR3

**Figure 4:**
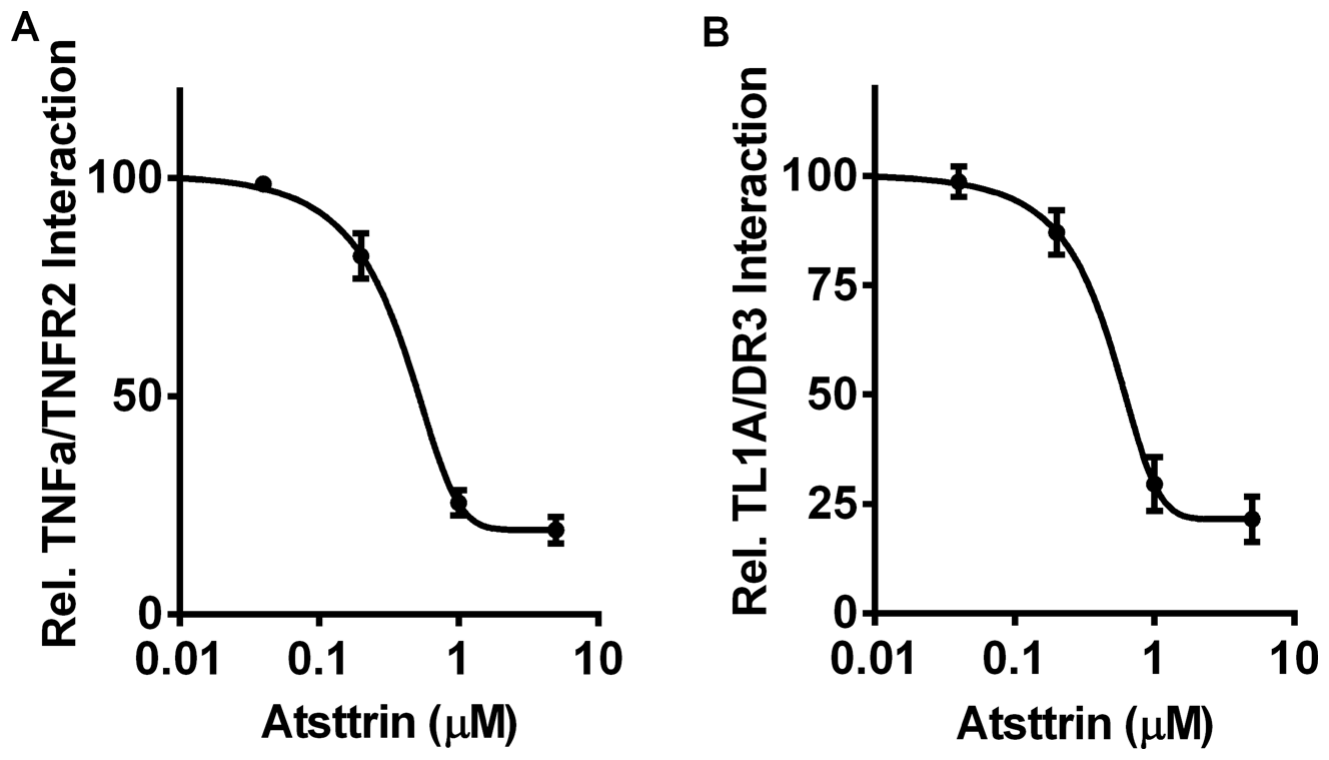
Atsttrin inhibits the binding of TL1A to DR3, similar to its inhibition of TNFa/ TNFR2 interaction. ELISA plate was coated with 100(A) or TL1A (B), and then 100 ng of TNFR2 or DR3 was added to each well respectively, in the presence of various Atsttrin, the bound TNFR2 or DR3 was detected by anti-TNFR2 or anti-DR3 antibody, followed by a secondary antibody conjugated with horseradish peroxidase.

Previous report showed that Atsttrin dose-dependently inhibited the binding of TNFa to TNFR through competing for binding to TNFR [3], together with the finding that Atsttrin also bound to DR3, led us to examine whether Atsttrin also affected the interaction between DR3 and TL1A, the only known ligand for DR3 [17]. In accordance with previous report [3], the Atsttrin we produced also inhibited the binding of TNFa to TNFR2 in a dose-dependent manner (Fig. 4A). Following the published procedure, the binding of DR3 to TL1A in the presence of various amounts of recombinant Atsttrin was measured using ELISA-based solid phase binding assay. As shown in Fig. 4B, Atsttrin demonstrated a dose-dependent inhibition of the interaction between TL1A and DR3.

### Atsttrin inhibits TL1A activity

**Figure 5:**
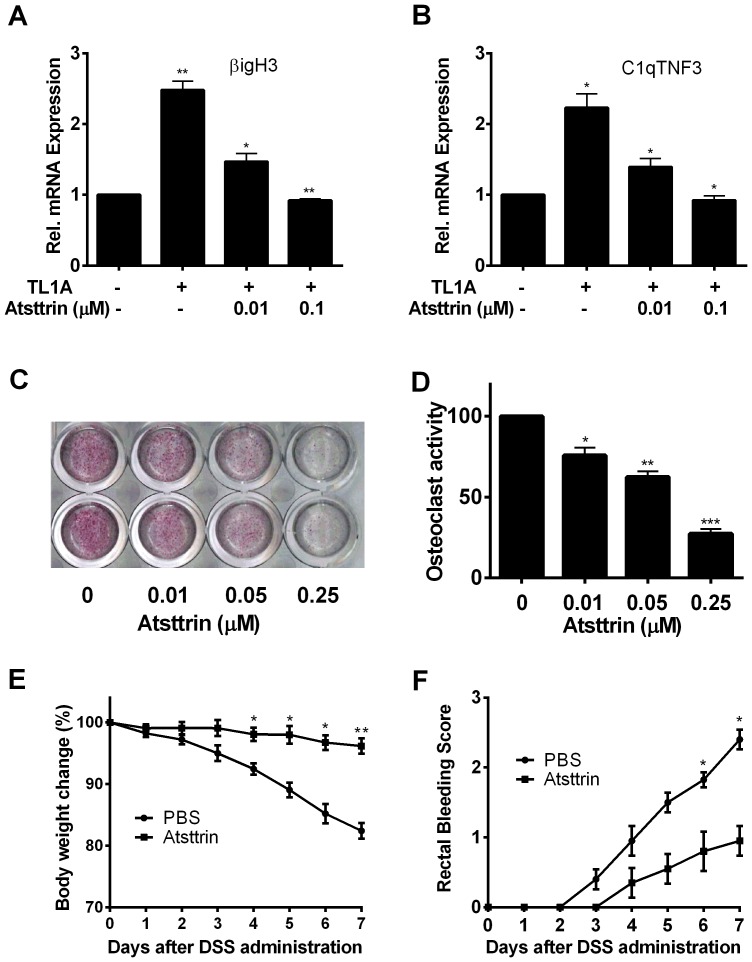
Atsttrin inhibits TL1A activity. **A, B. Atsttrin inhibits TL1A-activated gene expressions of βigH3 and C1qTNF3 in THP-1 cells.** Total RNA was extracted from THP-1 cells treated with 100 ng/ml of TL1A in the presence of various dose of Atsttrin, and then was reverse-transcribed to cDNA, expression level of βigH3 and C1qTNF3 was examined by quantitative real time PCR. **C. Atsttrin inhibits TL1A-enhanced osteoclastogenesis.** RAW264.7 cells were treated with 100 ng of TL1A and 35 ng/ml of RANKL in the presence of various dose of Atsttrin (as indicated), TRAP staining was then performed. **D. Quantitative assay of osteoclastogenesis.** Cells, treated as described in **C,** were washed twice with 0.9% sodium chloride; osteoclast activity was determined using 50 mM PNPP as substrate, the absorbance was measured at 540 nm. *p<0.05, **p<0.01, ***p<0.001. **E, F. Atsttrin prevented body weight loss and reduced bleeding score in DSS-induced colitis.** Mice challenged with 3% DSS were treated with either PBS or Atsttrin, and the body weight (**E**) and the bleeding score (**F**) were monitored daily.

Since Atsttrin was able to disturb the binding of TL1A to its receptor DR3, we then examined whether Atsttrin affected TL1A-activated gene expression. THP-1 cells were treated with 100 ng/ml TL1A in the presence of various dose of Atsttrin, mRNA expression levels of βigH3 and C1qTNF3, known to be the TL1A-induced genes [28], were examined by quantitative real time PCR. As expected, TL1A activated the expressions of βigH3 (Fig. 5A) and C1qTNF3 (Fig. 5B). Whereas this TL1A-mediated activation of gene expression was dose-dependently inhibited by Atsttrin (Fig. 5 A and B). In addition, Atsttrin also inhibited TL1A-mediated induction of these genes in TNFR1-/-:TNFR2-/- bone marrow-derived macrophages (BMDM) cells isolated from TNFR1 and TNFR2 double mutant mice (Fig. S2 in File S1), indicating that Atsttrin inhibition of TL1A-activated expressions of these genes is TNFR-independent.

It has been reported that TL1A is able to enhance RANKL-stimulated osteoclastogenesis in vitro [29], we then sought out to determine whether or not Atsttrin also affected TL1A activity in this assay. Briefly, RAW264.7 cells, a murine macrophage-like cell line widely used for in vitro osteoclastogenesis [3], were co-stimulated with 100 ng/ml TL1A and 35 ng/ml RANKL in the presence of various doses of Atsttrin, and osteoclastogenesis was monitored with TRAP staining. As shown in Fig. 5C, Atsttrin showed dose-dependent suppression of TL1A-enhanced osteoclastogenesis. Furthermore, Atsttrin inhibition of TL1A-enhanced osteoclastogenesis was also determined using quantitative assay (Fig. 5D). Collectively, these data clearly demonstrated that Atsttrin inhibited TL1A activity in these cells.

### Atsttrin prevented the body weight loss and bleeding in DSS-induced colitis

TL1A/DR3 has been implicated in the pathogenesis of inflammatory bowel diseases, including ulcerative colitis, and Crohn's disease [30]–[34], and Atsttrin has been shown to effectively attenuate pathology in inflammatory arthritis [3] and dermatitis [6] models, we next sought to determine whether recombinant Atsttrin was able to ameliorate colitis syndrome in DSS-induced mice model. The mice were subjected to induction of colitis by administration of 3% DSS water. Mice (n = 6) in Atsttrin group were injected with 50 μg Atsttrin every three days beginning at day 1 after DSS induction, whereas mice in control group (n = 6) were treated with PBS. Mice in PBS group suffered from significant body weight loss from day 4 to day 7, compared to the Atsttrin-treated group (**Fig. 5E**). Additionally, Atsttrin also effectively reduced bleeding observed in untreated mice with DSS-induced colitis (**Fig. 5F**).

## Discussion

PGRN and its derivative Atsttrin, were reported to bind to TNFR and inhibit TNFα activity in vitro, and poses the ability to suppress inflammation in vivo [1], [3], [6]. These findings draw great attention from the fields of inflammation research, since TNFα has been recognized as a master regulator of inflammation, and TNF inhibitors have been highly successful for treatment of several immune diseases including rheumatoid arthritis and Crohn's disease [9], [10]. In addition, these findings have been supported by recent publications from several laboratories [26], [35]–[42]. For instance, PGRN protected neuronal injury by inhibiting TNFα binding to the neutrophil, and in turn suppressing TNFα-induced neutrophil chemotaxis [38]. PGRN also played a protective role in atherosclerosis through suppressing TNFα-induced expression of ICAM-1 and VCAM-1 in endothelial cells [39]. PGRN blocked TNFα-triggered loss of the primary cilia in mesenchymal stromal cells through inhibiting NF-κB signaling [41]. PGRN antibodies entertained a proinflammatory environment in a subgroup of patients with psoriatic arthritis [42], and TNF-α-induced cytotoxicity assays demonstrated that the protective effects of PGRN were inhibited by serum containing PGRN antibodies [42]. TNFR2 pathway was found to be important for PGRN-mediated protection of lung inflammation [35] and for Atsttrin-mediated anti-inflammation in inflammatory arthritis [3]. It is also noted that the interaction of PGRN with TNFR was challenged by Chen et al. [43]. As described in the letter-to-editor concerning Chen's paper [43] (http://www.jneurosci.org/content/33/21/9202/reply#jneuro_el_111445), the demonstration of high-affinity interactions of PGRN/TNFR with surface plasmon resonance is highly dependent upon the type of chip used as well as the source of the recombinant PGRNs. Indeed, the behaviours of recombinant proteins used in Chen paper is puzzling, as data generated with their PGRN appears to be inconsistent. Recently, Jian et al. showed in detail that PGRN binds as TNFα to the 2^nd^ and 3^rd^ cysteine-rich domain in the extracellular portion of TNFR and that proper folding of PGRN is essential for this binding [26]. Our results that Atsttrin bound to DR3 led us to examine the interaction of PGRN with DR3. Solid phase binding demonstrated that PGRN also directly bound to DR3, similar to TNFR2 (**Fig. S1 in File S1**). Thus, our data also confirmed and extended the observations made by Tang et al. regarding the direct binding of PGRN and Atsttrin to TNFR [3], [26], and were in accordance with the reports from most laboratories [26], [35]–[42].

The fact that TNF inhibitors, such as antibodies or soluble TNFR proteins have been highly successful for treatment of several immune diseases, including rheumatoid arthritis [9], [10], led to great interest in other members of the superfamily as possible alternate or additional therapeutic targets for inflammatory and autoimmune disease. In this study we isolated DR3 as another member of TNFR super family to which Atsttrin binds as well in a screen based on yeast-two-hybrid system, followed by the confirmation using in vitro solid-phase binding assay (**Figs. 1** **,** **2**). In addition, Atsttrin dose-dependently inhibited TL1A-stimulated expressions of TL1A-target genes C1qTNF3 and βigH3 [28] (**Fig. 5**). Furthermore, Atsttrin effectively neutralized TL1A-promoted osteoclastogenesis in vitro (**Fig. 5**). Several lines of evidence both from experimental models and from clinical studies reveal that TL-1A and DR3 pathway is also critically involved in the pathogenesis of rheumatoid arthritis [17], [23]. A positive role for the TL1A/DR3 pathway in arthritis development has also been demonstrated in either DR3-deficient mice or by treating wild-type mice with blocking anti-TL1A [29]. Furthermore, recombinant TL1A injection aggravated collagen-induced arthritis in mice [44]. Recombinant Atsttrin was reported to effectively inhibit inflammation in several inflammatory arthritis models, including collagen antibody- and collagen-induced arthritis models, and TNF transgenic mice, through mediating TNF/TNFR signaling pathways [3]. Our finding that Atsttrin also binds to DR3 and inhibits TL1A activities, together with the facts that the TL1A/DR3 pathway also plays a crucial role in the pathogenesis of inflammatory arthritis, suggest that Atsttrin exerts its anti-inflammatory activities in inflammatory arthritis through, at least in part, suppressing both TNF/TNFR and TL-1A/DR3 inflammatory pathways. In addition, Atsttrin, similar to PGRN [3], [35], may also activate the protective TNFR2 signaling that also account for its therapeutic effects in preclinical animal models [3], [6].

In addition to be involved in the inflammatory arthritis, TL1A/DR3 has been implicated in the pathogenesis of gut inflammation [8], with polymorphisms of TL1A linked with inflammatory bowel diseases, ulcerative colitis, and Crohn's disease [30]–[32]. In addition, transgenic mice that constitutively express TL1A develop T cell-dependent inflammatory small bowel pathology [33], [34]. Our finding that Atsttrin ameliorated the pathology of DSS-induced colitis (**Fig. 5**) also supported the concept that TL1A/DR3 pathway plays an important role in the pathogenesis of inflammatory bowel diseases. The activity of TL1A has also been expanded to other inflammatory situations. For instance, mice deficient in DR3 or TL1A have significantly reduced numbers of autoreactive CD4 T cells and are impaired in displaying clinical disease symptoms in murine EAE models [14], [45]. The activity of TL1A in this case may result from modulating T cells that contribute to disease as well as osteoclasts that are responsive to TL1A stimulation [14], [45]. In addition, DR3-deficient mice, or wild-type mice injected with anti-TL1A, display reduced airway inflammation and mucus production in Th2-driven models of asthma [14], [46]. TL-1A/DR3 was also reported to regulate immunity to certain bacteria [47], tumors [48], and to maintains neurological function [49], [50]. Furthermore, both TL1A and DR3 have been implicated as mediators of atherosclerosis through promoting macrophage foam cell formation [51].

Similar to TNFα, PGRN also bound to the second and third cysteine-rich domains (CRD) in the extracellular portion of TNFR [26]. Like TNFR, DR3 also has four CRD in its extracellular domain, and although the crystal structure of DR3 has yet to be solved, the structural modeling predicts a similar structure to TNFR1 in which primary contacts with its ligand TL1A are in the 2^nd^ and 3^rd^ CRD [52], [53]. In addition, a mutation linked to rheumatoid arthritis is in a region critical for structural integrity of ligand–receptor complexes at the end of CRD3 [52], [53]. Interestingly, our data have demonstrated that the first three CRD domains of the extracellular potion of DR3, i.e. CRD1, CRD2 and CRD3, are all required for interacting with Atsttrin (**Fig. 3**). These data may provide the molecular mechanism underlying Atsttrin inhibition of TL1A/DR3 interaction.

In summary, Atsttrin, an engineered molecule derived from PGRN growth factor, was developed originally as a novel biologics for regulating TNFα/TNFR pathways and for treating TNFα-related conditions, with the special focus on inflammatory arthritis [3]. In this study Atsttrin was found to associate with TL1A/DR3 pathway as well, and it affected the binding and activity of TL1A in the in vitro and in vivo assays. These findings provide new insight into the in vivo anti-inflammatory and immunoregulatory action of Atsttrin, and present Atsttrin as a promising biologics for treating various kinds of diseases and conditions associated with TL-1A/DR3 pathways as well.

## Materials and Methods

### Cell culture, antibodies and reagents

RAW264.7 cells were grown in Dulbecco's modified Eagle's Medium supplemented with 10% fetal calf serum. THP-1 cells were cultured in RPMI1640 medium plus with 10% fetal calf serum. DR3 antibody(CAT# SC-7909)was purchased from Santa Cruz Biotechnology (Santa Cruz, CA), Recombinant protein hTL1A (Cat# 1319-TL-010) and the recombinant extracellular portion of hDR3 (Cat# 943-D3-050) were purchased from R&D system (Minneapolis, MN). The recombinant extracellular portions of TNFR2 (CAT# SRP3163) was purchased from SIGMA ALDRICH INC (St. Louis, MO). Recombinant hPGRN was purchased from Adipogen, San Diego, CA (Cat. No. AG-40A-0188).

### Expression Constructs

Yeast Two Hybrid System vectors pDBLeu and pPC86 were used for the expression of Atsttrin and extracellular domains of TNFRSF members respectively, for detecting the protein-protein interaction by Yeast Two Hybrid System. cDNA encoding Atsttrin was inserted into pDBLeu vector using the following primers: forward-5′ ACGCGTCGACGCCCCAGGCTTCCTGCTGTGAAG3′ and reverse-5′AAGAATGCGGCCGCTGGGATTGGACAGCAGCCCCA3′. Primers for sub-cloning extracellular domains of TNFRSF members into pPC86 vector were listed in Table1. Indicated numbers of amino acids of each TNFRSF member used for expression are as follows: hTNFR1 (AA23-212), mTNFR2 (AA23-258), hLTβR (AA1-224), mOX40 (AA1-165), mCD40 (AA1-191), hCD95 (AA17-173), hDcR3 (AA1-193), mCD27 (AA-141), mCD30 (AA1-325), m4-1BB (AA1-159), hDR4 (AA1-229), hDR5 (AA1-178), hDcR1 (AA1-149), hDcR2 (AA1-180), mRANK (AA30-213), mOPG (AA1-401), hTACI (AA1-104), hBAFFR (AA1-35), hHVEM (AA1-162), hNGFR (AA29-255), hBCMA (AA1-41), mGITR (AA1-142), mTROY (AA1-149), mDR6 (AA1-211), hDR3 (AA34-192), mXEDAR (AA1-118), hEDAR (AA1-148), hRELT (AA1-90).

**Table 1:** Primers for sub-cloning extracellular portions of TNFRSF members into pPC86.

Gene	Forward primer (5′-3′)	Reverse primer (5′-3′)
TNFR1	ATGGTCGACATACCCCTCAGGGGTTA	ATGGCGGCCGCCACTGTGGTGCCTGA
TNFR2	ATGCCATGGCAGTGCCCGCCCAGGTTGTCTTGAC	ATGGCGGCCGCGCCACCCTTGGTACTTTGTTCAA
LTβR	ATCGGTCGACCATGCTCCTGCCTTGGGCCACCT	AATTGCGGCCGCTCAGGTTCCTGACATCTCTGGGGGCAGT
OX40	ATGCGTCGACCATGTATGTGTGGGTTCAGCAGCC	ATGTGCGGCCGCTTACTCACAGACTGCGTCCAAGC
CD40	ATGCGTCGACCATGGTGTCTTTGCCTCGGC	ATGTGCGGCCGCTTACCGGGACTTTAATCCACAGA
CD95	ATCGGTCGACTAGATTATCGTCCAAAAGTGTTAATGC	AATTGCGGCCGCTCAGTTAGATCTGGATCCTTCCTCTTTG
DcR3	ATGGTCGACCATGAGGGCGCTGGAGGG	ATGGCGGCCGCTCAGCACAGGGTGTCATGGG
CD27	ATGCGTCGACCATGGCATGGCCACCTCCC	ATGTGCGGCCGCTTATGGTGGCTGTGGGCTCG
CD30	ATGCGTCGACCATGAGCGCCCTACTCACCG	ATGTGCGGCCGCTTAGGAAGGCAGCTCACAGATAGT
4-1BB	ATGCCATGGCAATGGGAAACAACTGTTACAACGTG	ATGTGCGGCCGCTTATCCACACACCACGTCCTTCT
DR4	ATGGTCGACCATGGCGCCACCACCAGC	ATGGCGGCCGCTCAACACTCGATGTCACTCCAGG
DR5	ATGCGTCGACCATGGAGCCTCCAGGACCCA	ATGTGCGGCCGCTTAACACTTCCGGTTTTCTCTGG
DcR1	ATGGTCGACCATGGCCCGGATCCCCAA	ATGGCGGCCGCTCAACACTGGATATCATCCCAGGA
DcR2	ATGGTCGACCATGGGACTTTGGGGACAAAG	ATGGCGGCCGCTCAGCACTTGATGTCACTCCG
RANK	GCATGTCGACCCAGGTCACTCCTCCATGCACCCA	TTAAGCGGCCGCTCAGGGCAGGTAAGCCTGGGCCTCCTTG
OPG	ATGCGTCGACCATGAACAAGTGGCTGTGCTGC	ATGCGCGGCCGCTTATAAGCAGCTTATTTTCACGGATT
TACI	ATGCGTCGACCATGGCTATGGCATTCTGCCC	ATGTGCGGCCGCTTAACAGAAGTGGGCACACTGCT
BAFFR	ATGGTCGACCATGAGGCGAGGGCCCCGGA	ATGGCGGCCGCTCAGCAGGCCACGCAGTGGCG
HVEM	ATGGTCGACCATGGAGCCTCCTGGAGACTG	ATGGCGGCCGCTCAACACAGGGTGTCCTGACTC
NGFR	ATCGGTCGACCAAGGAGGCATGCCCCACAGG	AATTGCGGCCGCTCAATAGACAGGGATGAGGTTGTCGGT
BCMA	ATGGTCGACCATGTTGCAGATGGCTGGGCA	ATGGCGGCCGCTCAACAATAACGCTGACATGTTAGA
GITR	ATGCGTCGACCATGGGGGCATGGGCCAT	ATGTGCGGCCGCTTAGATGCACACAGCATTGTGGG
TROY	ATGCGTCGACCATGGCACTCAAGGTCCTACCTCTAC	ATGTGCGGCCGCTTAACAGTGTGGTTCGTAGGGAGG
DR6	ATGCGTCGACCATGGGGACCCGGGCAAG	ATGTGCGGCCGCTTAACAGACGTTGTCTGTCTCCTTG
DR3	ATGGTCGACCGACTGTGCCGGTGACTTCCA	ATGGCGGCCGCTCAGGCACAGCGCTCTGGACA
XEDAR	ATGCGTCGACCATGGATTGTCAAGAGAATGAGTACCG	ATGTGCGGCCGCTTAACACTGAACCTCGGAAGAAGGA
EDAR	ATGGTCGACCATGGCCCATGTGGGGGAC	ATGGCGGCCGCTCAACATTCCTTGGTGTTGGG
RELT	ATGGTCGACCATGAAGCCAAGTCTGCTGTGC	ATGGCGGCCGCTCAACAGAGTGTATCTCGAGTTG

**Note:** The restriction enzyme cleavage sites used for subcloning are highlighted in bold and underlined.

Various deletion mutants of extracellular portion of hDR3, as indicated in Fig. 3A, were also amplified and constructed into pPC86 vector using *SalI/NotI* site, to detect their interactions with Atsttrin, the numbers of amino acids of each domain are as follows: CRD1 (AA34-71), CRD2 (AA72-115), CRD3 (AA116-163), CRD4 (AA164-192).

GST-fusion construct was prepared by inserting cDNA encoding Atsttrin into the multiple cloning site of pGEX-3X, a bacterial vector for expressing GST fusions with a Factor Xa site, using *BamHI* and *EcoRI* restriction sites and transformed into host *E. coli DH5a* to produce recombinant proteins. The following primers specific for Atsttrin are used: Forward-5′GCGGGATCCTGCCCCAGGCTTCCTGCTGTGAAG3′ and Reverse-5′GCGGAATTCTGGGATTGGACAGCAGCCCCA3′.

### Expression and purification of Atsttrin protein

The published protocol was essential followed [3]. Bacterial culture transformed with GST-Atsttrin fusion construct was incubated overnight at 37°C and diluted 1∶10 in fresh complex medium containing 100 μg/ml ampicillin. Continue incubation for another 2-3 hr (OD600  = 0.6–1.0). Fusion protein was purified on a Glutathione–Sepharose column, Atsttrin was released from GST-fusion protein by Factor Xa cleavage.

### Yeast two hybrid system

Yeast two hybrid system was used for detecting the interactions between Atsttrin and TNFRSF members. Atsttrin was fused to the Gal4 DNA binding domain and each TNFRSF member was fused to the Gal4 activation domain. Selected plasmids were co-transformed into yeast strain MAV203 and plated on the synthetic complete medium (SC medium) lacking leucine and tryptophan, X-Gal assay was performed to determine β-galactosidase phenotype.

### Quantitative assays for β-gal in liquid culture using ONPG

Quantitative assays for β-galactosidase (β-gal) activity in liquid cultures were performed using o-nitropenyl-β-D-galactopyranoside (ONPG) as a substrate. For each strain, three independent colonies were analyzed and triplicate samples for each colony. Isolated colony was incubated in 2.5 ml SC medium lacking leucine and tryptophan (SC-Leu-Trp) with shaking overnight at 30°C, 1 ml overnight culture was transferred into 5 ml YPD medium with a starting OD600 of about 0.5, and incubated for another 2–3 hours until OD600 = 1.0–1.5. Cells were collected and lysed by vortexing with glass bead, 700 ul 4 mg/ml ONPG in Z buffer (60 mM Na2HPO4, 40 mM NaH2PO4, 10 mM KCl and 1 mM MgSO4, PH 7.0) was added to each extract, and incubated in a 30°C waterbath. The reaction was stopped by adding 400 ul 1 M Na2CO3 to each reaction when a yellow color developed. Record the time and enzyme activity was measured at OD420.

### Solid phase binding assay

To examine the binding of Atsttrin to TNFR or DR3, or the binding of Progranulin to DR3, ELISA-based solid phase binding assay was performed. Briefly, various dose of Atsttrin or progranulin was coated onto an ELISA plate overnight, after blocking, biotinylated TNFR2 or DR3 was added to each well, bound protein was detected by adding avidin-HRP to each well and the absorbance was measured at OD 450 nm.

To examine the inhibition of Atsttrin on the TNFa/TNFR or TL1A/DR3 interaction, 100 ng of TNFa or TL1A was coated to an ELISA plate overnight, after blocking, various dose of Atsttrin was added to each well together with 100 ng of TNFR2 or DR3, respectively, and bound protein was detected by anti-TNFR2 or anti-DR3 antibody.

### Quantitative real time PCR

To examine the βigh3 and C1qTNF3 expression level in the THP-1 cells or TNFR1-/-:TNFR2-/- mouse BMDM cells after treatment with TL1A, quantitative real time PCR was performed. Cells in 6 well plates (4×106 cells/ml) were pretreated with various dose of Atsttrin for 30 min, and then stimulated with 100 ng/ml TL1A for 24 h. Total RNA was extracted from whole cells and reverse-transcribed to cDNA, real time PCR was performed using sequence-specific primers: forwar-5′GTACTTCACCAACTGCAAGCAGTGG3′, and reverse-5′ CGTAAAGGTTTGAGAGTGGTAGGGC3′ for human βigH3, forward-5′ AATCCCTGAGACCAGATGAGCTACC3′ and reverse-5′CCTTGGTAGCCTCGAAAGCTGTAGT3′ for human C1qTNF3, forwar-5′TACTTCACCAACTGCAAGCAGTGGT3′, and reverse-5′ GATGGTGAAGCTTCCGGGTCCC3′ for mouse βigH3, forward-5′ CATTCTGGGGCCAGTCTCCACA3′ and reverse-5′GGCCTAGGTCGCCTTTGTCTCCT3′ for mouse C1qTNF3.

### Osteoclastogenesis

RAW264.7 cells were plated 2×10^4^ cells/well in a 48-well dish in DMEM with 10% FBS, and cultured in a humidified atmosphere of 5% CO2 at 37°C. Cells were then treated with 100 ng/ml TL1A, 35 ng/ml RANKL plus various dose of Atsttrin, medium was changed every three days. Tatrate Resistance Acid Phospatase Staining was performed on day 5. For quantitative assay of osteoclastogenesis, cells were stimulated for 5 days as above; cells were then washed twice with 0.9% sodium chloride, 50 mM PNPP substrate solution was added to each well and incubated at 37°C for 3 hours, the absorbance was measured at 540 nm.

### Induction of colitis

DSS colitis was induced by addition of dextran sodium sulfate (DSS; 36,000–50,000 MW) to the drinking water for 5 days, then replaced DSS solution with normal water [54]. To determine whether Atsttrin could ameliorate the symptoms of colitis seen in DSS-challenged mice, 50 μg recombinant Atsttrin was injected intraperitoneally every three days beginning at day 1 after DSS induction. The disease progression was determined by body weight changes, the presence of rectal bleeding. The bleeding score was evaluated according to the reference [54].

## Supporting Information

File S1 **Figure S1 & S2.**
(PDF)
