# Supplementary material for: Progranulin-Derived Atsttrin Directly Binds to TNFRSF25 (DR3) and Inhibits TNF-Like Ligand 1A (TL1A) Activity
Source: PLoS One. 2014 Mar 20;9(3):e92743. doi: 10.1371/journal.pone.0092743 (PMC3961393; doi:10.1371/journal.pone.0092743)
Supplement: File S1 — Figure S1 & S2. (PDF) [file pone.0092743.s001.pdf]

Supplementary Data

Progranulin-Derived Atsttrin Directly Binds to TNFRSF25 (DR3) and Inhibits TNF-Like Ligand 1A (TL1A) Activity

Cui Liu[1], Xing-Xia Li[1], Wei Gao[2], Wen Liu[3], De-Shan Liu[4]

From [1]Department of Pediatric Surgery, Qilu Hospital of Shandong University, Jinan, China; [2]Department of Nursing, Qilu Hospital of Shandong University, Jinan, China; [3]Department of Biotechnology, Taishan Medical University, Taian, China; and [4]Department of Traditional Chinese Medicine, Qilu Hospital of Shandong University, Jinan, China

This PDF file includes:

Figs. S1 and S2

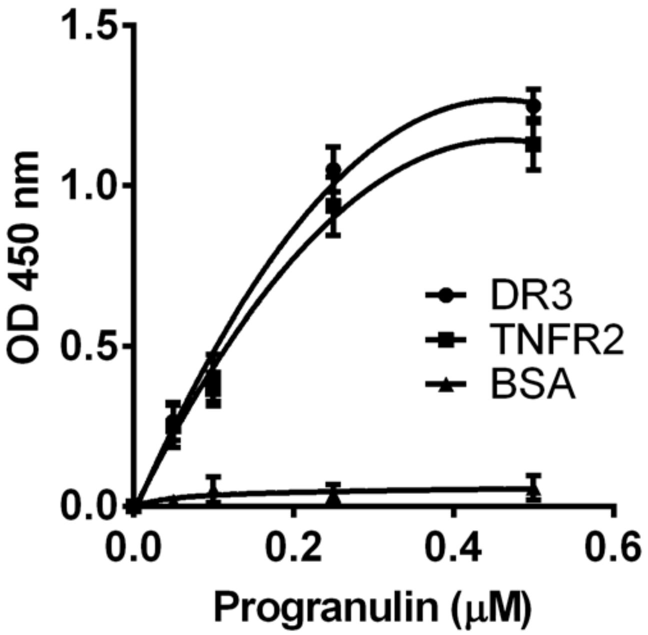

Figure S1. Progranulin directly binds to DR3, examined by solid phase binding assay. Various dose of progranulin was coated to ELISA plate, biotinylated DR3, TNFR2 (serving as a positive control) or BSA (serving as a negative control) was then added to each well, bound protein was detected by adding avidin-HRP to each well and the absorbance was measured at OD 450 nm.

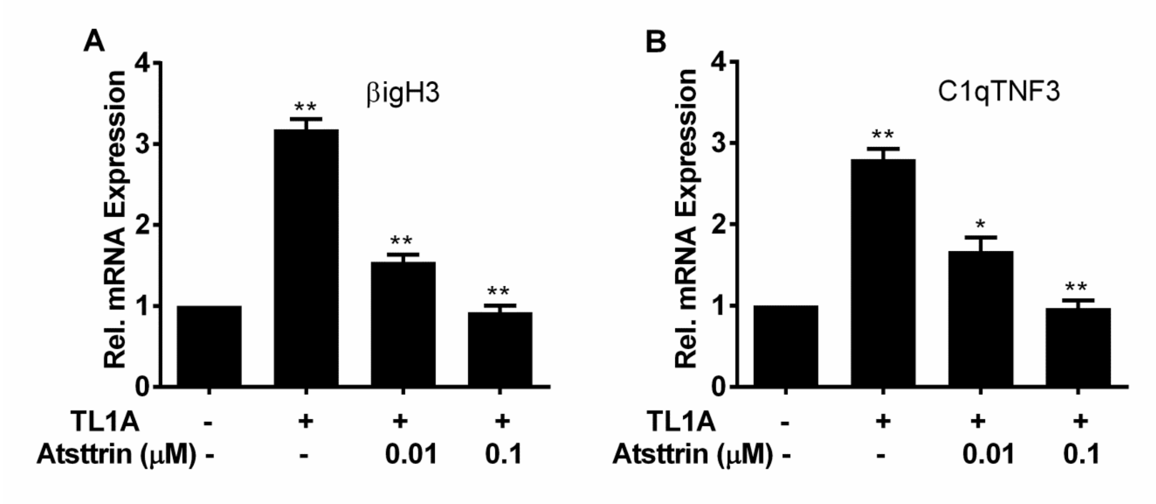

Figure S2. Atsttrin inhibits TL1A-mediated gene expressions of βigH3 and C1qTNF3 in TNFR1$^{-/-}$:TNFR2$^{-/-}$ BMDM cells. TNFR1$^{-/-}$:TNFR2$^{-/-}$ bone marrow-derived macrophages (BMDM) cells isolated from TNFR1 and TNFR2 double mutant mice were treated with 100 ng/ml of TL1A in the presence of various dose of Atsttrin. Total RNA was then extracted from cells and reverse-transcribed to cDNA, expression level of βigH3 and C1qTNF3 was examined by quantitative real time PCR.
